# Supplementary material for: A knowledge graph construction method for compliance review of water conservancy project reports
Source: PLoS One. 2026 Jan 12;21(1):e0339575. doi: 10.1371/journal.pone.0339575 (PMC12795383; doi:10.1371/journal.pone.0339575)
Supplement: S1 File — (DOCX) [file pone.0339575.s001.docx]

{"id":170,"text":"During water retaining, the crest elevation of the sluice shall not be lower than the sum of the normal water storage level of the sluice, the calculated wave height, and the safety freeboard of the normal water storage level.","entities":[{"id":28,"label":"obj","start_offset":0,"end_offset":14},{"id":29,"label":"cmp","start_offset":44,"end_offset":56},{"id":30,"label":"prop","start_offset":16,"end_offset":42},{"id":31,"label":"robj","start_offset":58,"end_offset":101},{"id":32,"label":"robj","start_offset":103,"end_offset":135},{"id":33,"label":"rprop","start_offset":137,"end_offset":140}],"relations":[],"Comments":[]}

{"id":171,"text":"During water retaining, the crest elevation of the sluice shall not be lower than the sum of the maximum water retaining level of the sluice, the calculated wave height, and the safety freeboard of the maximum water retaining level.","entities":[{"id":34,"label":"obj","start_offset":0,"end_offset":14},{"id":35,"label":"cmp","start_offset":44,"end_offset":56},{"id":36,"label":"prop","start_offset":16,"end_offset":42},{"id":37,"label":"robj","start_offset":58,"end_offset":107},{"id":38,"label":"robj","start_offset":109,"end_offset":147},{"id":39,"label":"rprop","start_offset":149,"end_offset":152}],"relations":[],"Comments":[]}

{"id":172,"text":"During water releasing, the crest elevation of the sluice shall not be lower than the sum of the design flood level and the safety freeboard of the design flood level.","entities":[{"id":40,"label":"obj","start_offset":0,"end_offset":14},{"id":41,"label":"cmp","start_offset":44,"end_offset":56},{"id":42,"label":"prop","start_offset":16,"end_offset":42},{"id":43,"label":"robj","start_offset":58,"end_offset":81},{"id":44,"label":"robj","start_offset":83,"end_offset":115},{"id":45,"label":"rprop","start_offset":117,"end_offset":120}],"relations":[],"Comments":[]}

{"id":173,"text":"During water releasing, the crest elevation of the sluice shall not be lower than the sum of the check flood level and the safety freeboard of the check flood level.","entities":[{"id":46,"label":"obj","start_offset":0,"end_offset":14},{"id":47,"label":"cmp","start_offset":44,"end_offset":56},{"id":48,"label":"prop","start_offset":16,"end_offset":42},{"id":49,"label":"robj","start_offset":58,"end_offset":80},{"id":50,"label":"robj","start_offset":82,"end_offset":114},{"id":51,"label":"rprop","start_offset":116,"end_offset":119}],"relations":[],"Comments":[]}

{"id":174,"text":"For Grade 1 sluices during water retaining, the safety freeboard of the normal water storage level shall not be less than 0.7m.","entities":[{"id":52,"label":"obj","start_offset":0,"end_offset":33},{"id":53,"label":"cmp","start_offset":70,"end_offset":82},{"id":54,"label":"prop","start_offset":35,"end_offset":68},{"id":55,"label":"robj","start_offset":84,"end_offset":88}],"relations":[],"Comments":[]}

{"id":175,"text":"For Grade 1 sluices during water retaining, the safety freeboard of the maximum water retaining level shall not be less than 0.5m.","entities":[{"id":56,"label":"obj","start_offset":0,"end_offset":33},{"id":57,"label":"cmp","start_offset":76,"end_offset":88},{"id":58,"label":"prop","start_offset":35,"end_offset":74},{"id":59,"label":"robj","start_offset":90,"end_offset":94}],"relations":[],"Comments":[]}

{"id":176,"text":"For Grade 1 sluices during water releasing, the safety freeboard of the design flood level shall not be less than 1.5m.","entities":[{"id":60,"label":"obj","start_offset":0,"end_offset":33},{"id":61,"label":"cmp","start_offset":70,"end_offset":82},{"id":62,"label":"prop","start_offset":35,"end_offset":68},{"id":63,"label":"robj","start_offset":84,"end_offset":89}],"relations":[],"Comments":[]}

{"id":177,"text":"For Grade 1 sluices during water releasing, the safety freeboard of the check flood level shall not be less than 1.0m.","entities":[{"id":64,"label":"obj","start_offset":0,"end_offset":33},{"id":65,"label":"cmp","start_offset":69,"end_offset":81},{"id":66,"label":"prop","start_offset":35,"end_offset":67},{"id":67,"label":"robj","start_offset":83,"end_offset":88}],"relations":[],"Comments":[]}

{"id":178,"text":"For Grade 2 sluices during water retaining, the safety freeboard of the normal water storage level shall not be less than 0.5m.","entities":[{"id":68,"label":"obj","start_offset":0,"end_offset":33},{"id":69,"label":"cmp","start_offset":70,"end_offset":82},{"id":70,"label":"prop","start_offset":35,"end_offset":68},{"id":71,"label":"robj","start_offset":84,"end_offset":88}],"relations":[],"Comments":[]}

{"id":179,"text":"For Grade 2 sluices during water retaining, the safety freeboard of the maximum water retaining level shall not be less than 0.4m.","entities":[{"id":72,"label":"obj","start_offset":0,"end_offset":33},{"id":73,"label":"cmp","start_offset":76,"end_offset":88},{"id":74,"label":"prop","start_offset":35,"end_offset":74},{"id":75,"label":"robj","start_offset":90,"end_offset":94}],"relations":[],"Comments":[]}

{"id":180,"text":"For Grade 2 sluices during water releasing, the safety freeboard of the design flood level shall not be less than 1.0m.","entities":[{"id":76,"label":"obj","start_offset":0,"end_offset":33},{"id":77,"label":"cmp","start_offset":70,"end_offset":82},{"id":78,"label":"prop","start_offset":35,"end_offset":68},{"id":79,"label":"robj","start_offset":84,"end_offset":89}],"relations":[],"Comments":[]}

{"id":181,"text":"For Grade 2 sluices during water releasing, the safety freeboard of the check flood level shall not be less than 0.7m.","entities":[{"id":80,"label":"obj","start_offset":0,"end_offset":33},{"id":81,"label":"cmp","start_offset":69,"end_offset":81},{"id":82,"label":"prop","start_offset":35,"end_offset":67},{"id":83,"label":"robj","start_offset":83,"end_offset":87}],"relations":[],"Comments":[]}

{"id":182,"text":"For Grade 3 sluices during water retaining, the safety freeboard of the normal water storage level shall not be less than 0.4m.","entities":[{"id":84,"label":"obj","start_offset":0,"end_offset":33},{"id":85,"label":"cmp","start_offset":70,"end_offset":82},{"id":86,"label":"prop","start_offset":35,"end_offset":68},{"id":87,"label":"robj","start_offset":84,"end_offset":88}],"relations":[],"Comments":[]}

{"id":183,"text":"For Grade 3 sluices during water retaining, the safety freeboard of the maximum water retaining level shall not be less than 0.3m.","entities":[{"id":88,"label":"obj","start_offset":0,"end_offset":33},{"id":89,"label":"cmp","start_offset":76,"end_offset":88},{"id":90,"label":"prop","start_offset":35,"end_offset":74},{"id":91,"label":"robj","start_offset":90,"end_offset":94}],"relations":[],"Comments":[]}

{"id":184,"text":"For Grade 3 sluices during water releasing, the safety freeboard of the design flood level shall not be less than 0.7m.","entities":[{"id":92,"label":"obj","start_offset":0,"end_offset":33},{"id":93,"label":"cmp","start_offset":70,"end_offset":82},{"id":94,"label":"prop","start_offset":35,"end_offset":68},{"id":95,"label":"robj","start_offset":84,"end_offset":87}],"relations":[],"Comments":[]}

{"id":185,"text":"For Grade 3 sluices during water releasing, the safety freeboard of the check flood level shall not be less than 0.5m.","entities":[{"id":96,"label":"obj","start_offset":0,"end_offset":33},{"id":97,"label":"cmp","start_offset":69,"end_offset":81},{"id":98,"label":"prop","start_offset":35,"end_offset":67},{"id":99,"label":"robj","start_offset":83,"end_offset":87}],"relations":[],"Comments":[]}

{"id":186,"text":"For Grade 4 sluices during water retaining, the safety freeboard of the normal water storage level shall not be less than 0.3m.","entities":[{"id":100,"label":"obj","start_offset":0,"end_offset":33},{"id":101,"label":"cmp","start_offset":70,"end_offset":82},{"id":102,"label":"prop","start_offset":35,"end_offset":68},{"id":103,"label":"robj","start_offset":84,"end_offset":88}],"relations":[],"Comments":[]}

{"id":187,"text":"For Grade 4 sluices during water retaining, the safety freeboard of the maximum water retaining level shall not be less than 0.2m.","entities":[{"id":104,"label":"obj","start_offset":0,"end_offset":33},{"id":105,"label":"cmp","start_offset":76,"end_offset":88},{"id":106,"label":"prop","start_offset":35,"end_offset":74},{"id":107,"label":"robj","start_offset":90,"end_offset":94}],"relations":[],"Comments":[]}

{"id":188,"text":"For Grade 4 sluices during water releasing, the safety freeboard of the design flood level shall not be less than 0.5m.","entities":[{"id":108,"label":"obj","start_offset":0,"end_offset":33},{"id":109,"label":"cmp","start_offset":70,"end_offset":82},{"id":110,"label":"prop","start_offset":35,"end_offset":68},{"id":111,"label":"robj","start_offset":84,"end_offset":87}],"relations":[],"Comments":[]}

{"id":189,"text":"For Grade 4 sluices during water releasing, the safety freeboard of the check flood level shall not be less than 0.4m.","entities":[{"id":112,"label":"obj","start_offset":0,"end_offset":33},{"id":113,"label":"cmp","start_offset":69,"end_offset":81},{"id":114,"label":"prop","start_offset":35,"end_offset":67},{"id":115,"label":"robj","start_offset":83,"end_offset":87}],"relations":[],"Comments":[]}

{"id":190,"text":"For Grade 5 sluices during water retaining, the safety freeboard of the normal water storage level shall not be less than 0.3m.","entities":[{"id":116,"label":"obj","start_offset":0,"end_offset":33},{"id":117,"label":"cmp","start_offset":70,"end_offset":82},{"id":118,"label":"prop","start_offset":35,"end_offset":68},{"id":119,"label":"robj","start_offset":84,"end_offset":88}],"relations":[],"Comments":[]}

{"id":191,"text":"For Grade 5 sluices during water retaining, the safety freeboard of the maximum water retaining level shall not be less than 0.2m.","entities":[{"id":120,"label":"obj","start_offset":0,"end_offset":33},{"id":121,"label":"cmp","start_offset":76,"end_offset":88},{"id":122,"label":"prop","start_offset":35,"end_offset":74},{"id":123,"label":"robj","start_offset":90,"end_offset":94}],"relations":[],"Comments":[]}

{"id":192,"text":"For Grade 5 sluices during water releasing, the safety freeboard of the design flood level shall not be less than 0.5m.","entities":[{"id":124,"label":"obj","start_offset":0,"end_offset":33},{"id":125,"label":"cmp","start_offset":70,"end_offset":82},{"id":126,"label":"prop","start_offset":35,"end_offset":68},{"id":127,"label":"robj","start_offset":84,"end_offset":87}],"relations":[],"Comments":[]}

{"id":193,"text":"For Grade 5 sluices during water releasing, the safety freeboard of the check flood level shall not be less than 0.4m.","entities":[{"id":128,"label":"obj","start_offset":0,"end_offset":33},{"id":129,"label":"cmp","start_offset":69,"end_offset":81},{"id":130,"label":"prop","start_offset":35,"end_offset":67},{"id":131,"label":"robj","start_offset":83,"end_offset":87}],"relations":[],"Comments":[]}

{"id":194,"text":"For sluices located on flood control dikes, their crest elevation shall not be lower than the top elevation of the flood control dike.","entities":[{"id":132,"label":"obj","start_offset":0,"end_offset":31},{"id":133,"label":"cmp","start_offset":51,"end_offset":63},{"id":134,"label":"prop","start_offset":33,"end_offset":49},{"id":135,"label":"robj","start_offset":65,"end_offset":92}],"relations":[],"Comments":[]}

{"id":195,"text":"For sluices located on tide protection dikes, their crest elevation shall not be lower than the top elevation of the tide protection dike.","entities":[{"id":136,"label":"obj","start_offset":0,"end_offset":32},{"id":137,"label":"cmp","start_offset":52,"end_offset":64},{"id":138,"label":"prop","start_offset":34,"end_offset":50},{"id":139,"label":"robj","start_offset":66,"end_offset":94}],"relations":[],"Comments":[]}

{"id":196,"text":"The bottom elevation of the beams (slabs) of the service bridge shall be 0.5m or more above the maximum flood level.","entities":[{"id":140,"label":"obj","start_offset":0,"end_offset":14},{"id":141,"label":"cmp","start_offset":58,"end_offset":71},{"id":142,"label":"prop","start_offset":16,"end_offset":56},{"id":143,"label":"robj","start_offset":73,"end_offset":98}],"relations":[],"Comments":[]}

{"id":197,"text":"The bottom elevation of the beams (slabs) of the maintenance bridge shall be 0.5m or more above the maximum flood level.","entities":[{"id":144,"label":"obj","start_offset":0,"end_offset":14},{"id":145,"label":"cmp","start_offset":64,"end_offset":77},{"id":146,"label":"prop","start_offset":16,"end_offset":62},{"id":147,"label":"robj","start_offset":79,"end_offset":104}],"relations":[],"Comments":[]}

{"id":198,"text":"The bottom elevation of the beams (slabs) of the traffic bridge shall be 0.5m or more above the maximum flood level.","entities":[{"id":148,"label":"obj","start_offset":0,"end_offset":14},{"id":149,"label":"cmp","start_offset":60,"end_offset":73},{"id":150,"label":"prop","start_offset":16,"end_offset":58},{"id":151,"label":"robj","start_offset":75,"end_offset":100}],"relations":[],"Comments":[]}

{"id":199,"text":"The seepage control length shall not be less than the product of the seepage path coefficient and the maximum water level difference between the upstream and downstream.","entities":[{"id":152,"label":"obj","start_offset":0,"end_offset":19},{"id":153,"label":"cmp","start_offset":22,"end_offset":34},{"id":154,"label":"prop","start_offset":0,"end_offset":21},{"id":155,"label":"robj","start_offset":36,"end_offset":59},{"id":156,"label":"robj","start_offset":61,"end_offset":110},{"id":157,"label":"rprop","start_offset":112,"end_offset":118}],"relations":[],"Comments":[]}

{"id":200,"text":"For loam foundations without filter layers, the allowable seepage path coefficient shall not be less than 4.","entities":[{"id":158,"label":"obj","start_offset":0,"end_offset":28},{"id":159,"label":"cmp","start_offset":60,"end_offset":72},{"id":160,"label":"prop","start_offset":30,"end_offset":58},{"id":161,"label":"robj","start_offset":74,"end_offset":75}],"relations":[],"Comments":[]}

{"id":201,"text":"For clay foundations without filter layers, the allowable seepage path coefficient shall not be less than 3.","entities":[{"id":162,"label":"obj","start_offset":0,"end_offset":28},{"id":163,"label":"cmp","start_offset":60,"end_offset":72},{"id":164,"label":"prop","start_offset":30,"end_offset":58},{"id":165,"label":"robj","start_offset":74,"end_offset":75}],"relations":[],"Comments":[]}

{"id":202,"text":"For silty sand foundations with filter layers, the allowable seepage path coefficient shall not be less than 9.","entities":[{"id":166,"label":"obj","start_offset":0,"end_offset":31},{"id":167,"label":"cmp","start_offset":63,"end_offset":75},{"id":168,"label":"prop","start_offset":33,"end_offset":61},{"id":169,"label":"robj","start_offset":77,"end_offset":78}],"relations":[],"Comments":[]}

{"id":203,"text":"For fine sand foundations with filter layers, the allowable seepage path coefficient shall not be less than 7.","entities":[{"id":170,"label":"obj","start_offset":0,"end_offset":30},{"id":171,"label":"cmp","start_offset":62,"end_offset":74},{"id":172,"label":"prop","start_offset":32,"end_offset":60},{"id":173,"label":"robj","start_offset":76,"end_offset":77}],"relations":[],"Comments":[]}

{"id":204,"text":"For medium sand foundations with filter layers, the allowable seepage path coefficient shall not be less than 5.","entities":[{"id":174,"label":"obj","start_offset":0,"end_offset":31},{"id":175,"label":"cmp","start_offset":63,"end_offset":75},{"id":176,"label":"prop","start_offset":33,"end_offset":61},{"id":177,"label":"robj","start_offset":77,"end_offset":78}],"relations":[],"Comments":[]}

{"id":205,"text":"For coarse sand foundations with filter layers, the allowable seepage path coefficient shall not be less than 4.","entities":[{"id":178,"label":"obj","start_offset":0,"end_offset":31},{"id":179,"label":"cmp","start_offset":63,"end_offset":75},{"id":180,"label":"prop","start_offset":33,"end_offset":61},{"id":181,"label":"robj","start_offset":77,"end_offset":78}],"relations":[],"Comments":[]}

{"id":206,"text":"For medium gravel foundations with filter layers, the allowable seepage path coefficient shall not be less than 3.","entities":[{"id":182,"label":"obj","start_offset":0,"end_offset":33},{"id":183,"label":"cmp","start_offset":65,"end_offset":77},{"id":184,"label":"prop","start_offset":35,"end_offset":63},{"id":185,"label":"robj","start_offset":79,"end_offset":80}],"relations":[],"Comments":[]}

{"id":207,"text":"For fine gravel foundations with filter layers, the allowable seepage path coefficient shall not be less than 3.","entities":[{"id":186,"label":"obj","start_offset":0,"end_offset":32},{"id":187,"label":"cmp","start_offset":64,"end_offset":76},{"id":188,"label":"prop","start_offset":34,"end_offset":62},{"id":189,"label":"robj","start_offset":78,"end_offset":79}],"relations":[],"Comments":[]}

{"id":208,"text":"For coarse gravel mixed with pebble foundations with filter layers, the allowable seepage path coefficient shall not be less than 2.5.","entities":[{"id":190,"label":"obj","start_offset":0,"end_offset":44},{"id":191,"label":"cmp","start_offset":76,"end_offset":88},{"id":192,"label":"prop","start_offset":46,"end_offset":74},{"id":193,"label":"robj","start_offset":90,"end_offset":93}],"relations":[],"Comments":[]}

{"id":209,"text":"For light silty sandy loam foundations with filter layers, the allowable seepage path coefficient shall not be less than 7.","entities":[{"id":194,"label":"obj","start_offset":0,"end_offset":43},{"id":195,"label":"cmp","start_offset":75,"end_offset":87},{"id":196,"label":"prop","start_offset":45,"end_offset":73},{"id":197,"label":"robj","start_offset":89,"end_offset":90}],"relations":[],"Comments":[]}

{"id":210,"text":"For light sandy loam foundations with filter layers, the allowable seepage path coefficient shall not be less than 5.","entities":[{"id":198,"label":"obj","start_offset":0,"end_offset":36},{"id":199,"label":"cmp","start_offset":68,"end_offset":80},{"id":200,"label":"prop","start_offset":38,"end_offset":66},{"id":201,"label":"robj","start_offset":82,"end_offset":83}],"relations":[],"Comments":[]}

{"id":211,"text":"For loam foundations with filter layers, the allowable seepage path coefficient shall not be less than 3.","entities":[{"id":202,"label":"obj","start_offset":0,"end_offset":29},{"id":203,"label":"cmp","start_offset":61,"end_offset":73},{"id":204,"label":"prop","start_offset":31,"end_offset":59},{"id":205,"label":"robj","start_offset":75,"end_offset":76}],"relations":[],"Comments":[]}

{"id":212,"text":"For clay foundations with filter layers, the allowable seepage path coefficient shall not be less than 2.","entities":[{"id":206,"label":"obj","start_offset":0,"end_offset":29},{"id":207,"label":"cmp","start_offset":61,"end_offset":73},{"id":208,"label":"prop","start_offset":31,"end_offset":59},{"id":209,"label":"robj","start_offset":75,"end_offset":76}],"relations":[],"Comments":[]}

{"id":213,"text":"The intersection angle between the center line of the intake sluice or diversion sluice and the center line of the river (canal) should be less than 30°.","entities":[{"id":210,"label":"obj","start_offset":0,"end_offset":24},{"id":211,"label":"cmp","start_offset":101,"end_offset":112},{"id":212,"label":"prop","start_offset":26,"end_offset":99},{"id":213,"label":"robj","start_offset":114,"end_offset":118}],"relations":[],"Comments":[]}

{"id":214,"text":"The segment length on rock foundations should not exceed 20m.","entities":[{"id":214,"label":"obj","start_offset":0,"end_offset":17},{"id":215,"label":"cmp","start_offset":34,"end_offset":45},{"id":216,"label":"prop","start_offset":19,"end_offset":32},{"id":217,"label":"robj","start_offset":47,"end_offset":51}],"relations":[],"Comments":[]}

{"id":215,"text":"The segment length on soil foundations should not exceed 35m.","entities":[{"id":218,"label":"obj","start_offset":0,"end_offset":17},{"id":219,"label":"cmp","start_offset":33,"end_offset":44},{"id":220,"label":"prop","start_offset":19,"end_offset":31},{"id":221,"label":"robj","start_offset":46,"end_offset":51}],"relations":[],"Comments":[]}

Minimal data
